# Immunosuppression in Patients with Chronic Hepatitis B

**DOI:** 10.1007/s11901-014-0238-2

**Published:** 2014-06-21

**Authors:** Anil Seetharam, Robert Perrillo, Robert Gish

**Affiliations:** 1 Banner Transplant and Advanced Liver Disease Center, Phoenix, AZ USA; 2 Hepatology Division, Baylor University Medical Center, Dallas, TX USA; 3 St. Joseph’s Hospital Medical Center/Liver Center, Phoenix, AZ USA; 4 University of Arizona College of Medicine, Phoenix, AZ USA; 5 6022 La Jolla Mesa Drive, San Diego, CA 92037 USA; 6 Department of Internal Medicine, University of Texas Southwestern Medical School, Dallas, TX USA

**Keywords:** Hepatitis B, Rituximab, HBV DNA, Immunosuppression, Chemotherapy, Reactivation, Lymphoma, Prophylactic therapy, Entecavir

## Abstract

After hepatitis B virus (HBV) infection, HBV DNA persists in minute amounts in hepatocyte nuclei even in individuals with “resolved” infection. Viral replication and development of liver disease depend on the balance between viral mechanisms promoting persistence and host immune control. Patients with active or inactive disease or resolved HBV infection are at risk for reactivation with immunosuppressive therapy use. HBV reactivation varies from a clinically asymptomatic condition to one associated with acute liver failure and death. We review recent studies on HBV reactivation during immunomodulatory therapies for oncologic, gastroenterological, rheumatic, and dermatologic disorders. Risk calculation should be determined through HBV screening and assessment of immunosuppressive therapy potency. We also discuss monitoring for reactivation, prophylactic antiviral therapy, and treatment of reactivation. Prophylactic antiviral treatment is needed for all HBsAg carriers and selected patients who have anti-HBc without HBsAg and is critical for preventing viral reactivation and improving outcomes.

## Introduction

Over half of the world’s population has been exposed to hepatitis B virus (HBV), and it is generally estimated that there are 350 million chronic carriers worldwide [1, 2]. HBV infection is usually denoted by the detection of hepatitis B surface antigen (HBsAg) in serum, while clearance of HBsAg is generally considered consistent with resolution of active infection. The vast majority of people with serological recovery from HBV infection (HBsAg-negative, antibody to hepatitis B core antigen (anti-HBc)-positive, with or without hepatitis B surface antibody [anti-HBs]) have undetectable HBV DNA in serum, yet HBV DNA persists in minute amounts in the nuclei of hepatocytes [3]. In individuals who do not clear the infection and progress to develop chronic HBV infection (HBsAg-positive and anti-HBc-positive), serum HBV DNA levels vary greatly from undetectable (<20 international units [IU]/ml) to >1,000,000,000 (>9 log10) IU/ml [4]. This broad range of serum HBV DNA reflects a balance between viral replication fitness and host control defenses, namely innate and adaptive immune system activation [5, 6].

Immunosuppression can alter this balance enough to induce the clinical entity of HBV reactivation (HBVr). Reactivation is best characterized as a virologic event in which there is a sudden increase in viral replication due to loss of immune control. Frequently, although not always, there is concomitant evidence of inflammatory liver disease, with an elevation in serum aminotransferase levels and, in severe cases, elevation of bilirubin level. While reactivation can also occur spontaneously as part of the natural history of chronic HBV infection, the most common clinical setting involves the use of chemotherapeutic or immunosuppressive drug therapy [7]. Reactivation during cancer chemotherapy has been well recognized but has also been reported with most immunosuppressive agents. Such agents are now often used in hematologic, gastrointestinal, rheumatic, dermatologic, and pulmonary diseases, as well as in transplantation (solid organ and stem cell). Inhibitors of interleukins 17 and 23 are currently under development and are likely to further increase rheumatology and dermatology patient exposure [8]. Thus, drug-induced HBVr will become a more common entity facing a broader range of medical specialists.

While the particular immune effector systems that are inhibited vary between drugs, all inhibit adaptive immunity to HBV which renders the chronically infected HBV patient with HBsAg(+) status or occult infection susceptible to reactivation [9]. It is not well understood why some patients demonstrate reactivation during immunosuppressive drug therapy whereas others with similar virologic and biochemical features fail to do so; nor is it understood what determines such a broad spectrum of clinical severity, from asymptomatic with minimal if any ALT increase to severe or fulminant. However, insights have been gained from recent case series that identify patient and viral factors that increase the likelihood of reactivation [10, 11]. Experience with newer immunosuppressive therapies has provided a better means of identifying agents that present a risk for reactivation [12, 13].

## Mechanisms of Reactivation

Once infected with HBV, patients harbor covalently closed circular (ccc) HBV DNA and pre-genomic viral transcripts in hepatocyte nuclei forever [14]. Even with serologic resolution of infection (with loss of HBsAg, undetectable serum HBV DNA, and appearance of anti-HBs) ccc HBV DNA can remain in hepatocytes and other cells for the life of the patient [15]. Rehermann et al. reported HBV cccDNA to be present in hepatocytes and circulating peripheral mononuclear cells following clinically resolved infection [16]. Even patients with isolated anti-HBc status (presence of anti-HBc without HBsAg or anti-HBs) who have “cleared” infection in the remote past also harbor minute quantities of HBV cccDNA in a small minority of hepatocytes and remain at risk of HBVr. During a period of viral latency, HBV replication in the liver or in peripheral mononuclear cells is controlled through effector arms of adaptive immunity, including HBV-specific CD4+ helper T cells, HBV-specific CD8+ cytotoxic T cells, appropriately primed B cells which can serve as antigen presenting cells, and cytokines such as interferon gamma and tumor necrosis factor (TNF) alpha [17]. When host adaptive immunity is compromised by immunosuppressive therapy directed against either T cells or B cells, there is resultant loss of immune control allowing for increased HBV replication. Moreover, biochemical flares sometimes occur once cytotoxic T cell-mediated responses are restored after completion of therapy (immune reconstitution) [18].

Non-immune pathways have also been implicated in HBVr. In HBV-infected transgenic mice, irradiation of the liver plus interleukin-6 therapy caused in vitro HBV replication through STAT3 signaling [19]. HBVr can also occur directly with steroid usage via stimulation of a glucocorticoid-responsive element in the HBV genome which leads to upregulation of HBV gene expression [20]. Viral factors that have been associated with sudden increases in HBV replication and immunologic flares during HBeAg-negative hepatitis B include the HBV precore mutation G1896A and the basal core promoter mutation T1762/A1764 [21, 22].

## Clinical Manifestations

Reactivation of HBV has a heterogeneous clinical presentation, ranging from asymptomatic and subclinical to severe acute liver failure and death. HBVr during cancer chemotherapy is often conceptualized to occur in phases. During the first phase, increasing serum HBV DNA levels occur. This can be accompanied by the reappearance of HBsAg [23]. Aminotransferases may or may not be elevated and the patient is often asymptomatic. The second phase begins days to weeks later when elevation in aminotransferase levels due to cellular immunity rebound are observed in response to further increase in viral replication and even higher serum HBV DNA. Clinical symptoms commonly encountered in hepatitis (fatigue, malaise, and jaundice) may become evident. During this phase, progression to acute liver failure and death may occur. With cancer chemotherapy, this second phase is thought to be explained by the immune reconstitution that occurs during the interval between courses of treatment; although this hepatitis flare frequently occurs after the first few cycles of chemotherapy, it can occur at any time [24, 25]. In severe HBVr with progressive hepatocyte injury and liver failure indicators of poor prognosis include jaundice, encephalopathy, ascites, elevated bilirubin levels or increased INR and renal failure as a late phase event. Liver transplantation can be considered; however, many patients are disqualified on the basis of their underlying disease (e.g., malignancy). In many patients, there is a third phase in which hepatic injury from HBVr either resolves spontaneously or following discontinuation of immunosuppressive therapy or initiation of antiviral therapy [26].

## Nomenclature

There is great need for standardized nomenclature and definition of HBVr. Varied definitions have been used to define the virologic event (de novo appearance of HBV DNA in a patient previously known to be negative; tenfold increase in HBV DNA over previous levels; or even HBV DNA >20,000 IU at the time of a sudden elevation of the aminotransferases). Many reports indicate that an increase in serum aminotransferases should be part of the definition; however, abnormalities of ALT or AST may not be observed [27]. In 2013, a consensus conference, the American Association for the Study of Liver Diseases (AASLD) Emerging Trends Conference, was convened by AASLD on HBVr [28]. An important part of the agenda was proposing standardized nomenclature to facilitate more accurate reporting and collaboration among practitioners from varied specialties. It was proposed that reactivation of HBV replication be defined as an increase in HBV replication (≥2 log increase from baseline level) or de novo appearance of HBV DNA in a patient in whom previous testing found it to be non-detectable. Reactivation of past hepatitis B can be further defined as reappearance of HBsAg (in those who were previously documented to be negative) or appearance of HBV DNA alone in the absence of HBsAg. Irrespective of the presenting virologic and serologic features, reactivation can vary from an asymptomatic condition to one that is life threatening with acute liver failure and possible need for transplantation.

## Risk Factors for Reactivation

Information from case series of chronically infected HBV patients has identified a number of risk factors for reactivation. These can be characterized as patient factors, viral factors, underlying diseases, type of transplantation as well as the type and intensity of immunosuppression [29–31].

## Patient Factors

Male sex and younger patient age have been associated with HBVr. In a recent study of 78 HBsAg-positive patients with various cancers receiving chemotherapeutic regimens, approximately 30 % of males had reactivation compared with 10 % of females [32]. It is unclear if gender-related differences in adaptive HBV-related immunity exist or if the increased risk with male sex relates to gender-based differences in incidence of diseases necessitating immunosuppressive therapy.

## Viral Factors

HBV replication status (prior to initiation of immunosuppressive therapy) has been identified as a risk factor. Chronically infected patients with HBsAg-positivity have a higher risk of reactivation compared with anti-HBc-positive patients who are HBsAg-negative. In HBsAg-positive patients, levels of HBV DNA prior to therapy are associated with risk of reactivation, with those having relatively high levels (>2000 IU) at higher risk compared with those having lower levels of HBV DNA [33]. The association between HBV DNA levels and risk of reactivation is likely also true for anti-HBc positive patients [34]. In patients who are HBsAg-negative and anti-HBc-positive, anti-HBs level is thought to be a factor as well, with those having undetectable anti-HBs level at the onset of immunosuppressive therapy and those who have loss of anti-HBs during immunosuppressive therapy at increased risk for reactivation [35, 36]. The association between the virologic and serologic status of the host and reactivation risk is likely to be explained by a spectrum of immunologic control over HBV, with HBeAg-positive, highly viremic HBsAg carriers at one extreme and non-viremic, anti-HBc/anti-HBs-positive patients at the other. Seen this way, it can be anticipated that the HBsAg carrier would be more likely to reactivate with mild to moderate immunosuppressive drug therapy (e.g., certain TNF-alpha inhibitors) whereas those positive for anti-HBc generally can be anticipated to have a much lower risk unless very intensive immunosuppressive treatments are given such as **R**ituximab combined with **C**yclophosphamide, doxorubicin hydrochloride/**H**ydroxydaunomycin, vincristine sulfate/**O**ncovin, and **P**rednisone (R-CHOP) for lymphoma or leukemia.

## Underlying Disease

The highest incidence of HBVr has been observed in patients receiving chemotherapy for hematologic malignancies, particularly lymphomas treated with R-CHOP. Whether lymphoma per se is associated with increased risk for HBVr is unknown. Both HBV and HCV are known to be chronic viruses leading to persistent B-cell activation and potential lymphomagenesis. Evidence exists to support direct infection of B cells and activation of signaling favoring tumorigenesis as well [37, 38]. This frequent association may be related to a higher prevalence of HBV infection among patients with lymphoma compared with controls [39] or may be a function of the intense immunosuppressive regimen used, particularly in regimens containing rituximab (Rituxan, Genentech, South San Francisco, CA) [40••]. Case series of those receiving chemotherapy for lymphoma have shown that reactivation often occurs after the second or third course of chemotherapy but can occur at any time [41•, 42].

HBVr has also been recorded in patients undergoing chemotherapy for solid tumors. Of the solid tumors, breast cancer is most commonly associated with HBVr, and studies estimate the rate of reactivation in HBsAg-positive patients with breast cancer to be 25–40 % [43, 44]. The high incidence of HBVr in patients with breast cancer has been attributed to the concomitant use of anthracyclines and corticosteroids. Both drugs have been shown to increase HBV DNA transcription. It has been shown in vitro that anthracyclines may promote transcription through increased HBV secretion from hepatoblasts [45] and steroids are known to increase viral transcription through a steroid responsive element in the viral genome [20]. HBVr has also been reported in patients treated for colon, lung, and head and neck cancers, albeit at an overall lower incidence (10-30 %) compared with breast [46, 47].

## Immunosuppression Intensity

Intensity of immunosuppression has often been implicated as a risk factor for HBVr [48]. When used in combination with other immunosuppressive agents for treatment of lymphoma, corticosteroids have been shown to increase the risk of reactivation. In one report, 49 HBsAg-positive patients with non-Hodgkin lymphoma were randomly assigned to receive chemotherapy with or without prednisolone. Reactivation occurred in 72 % of those who received prednisolone compared with only 38 % of those who did not [49]. Studies have also compared identical chemotherapy regimens for lymphoma with and without rituximab. In one study of 46 HBsAg-negative, anti-HBc-positive patients with lymphoma, 24 % of patients treated with R-CHOP had HBVr compared with none of those treated with only CHOP [50]. Evidence from these and other studies seem to support either an additive or synergistic effect of multiple immunosuppressive agents on risk of HBVr [51]. Unfortunately, this means that complex chemotherapy regimens often provide the best cancer responses but also a greater risk of HBV reactivation.

## Immunosuppressive Agents

Various immunosuppressive agents, targeting diverse effectors of the immune response, are used in oncologic, gastrointestinal, rheumatologic, and dermatologic diseases. Depending upon dose, combination, and patient risk factors, their use can lead to HBVr.

### Corticosteroids

Prednisone is a mainstay in some chemotherapeutic regimens and an important agent for inducing remission in inflammatory bowel disease. HBVr might partially be explained by glucocorticoid-associated suppression of T cell immune control. It is not known whether there is a threshold dose or duration of corticosteroid use above which the risk of HBVr increases but 2-4 weeks of low-dose therapy such as is used in asthma is not thought to present substantial risk. Reactivation has been reported in patients receiving corticosteroids alone for varying indications [52, 53], and appears to be at least additive when given in combination with other immunosuppressants.

### Anti-Metabolites

Anti-metabolic agents interfering with nucleic acid synthesis are often used in the treatment of inflammatory bowel disease and rheumatoid arthritis (RA). Isolated cases of HBVr have been reported in association with methotrexate and azathioprine [54]. In addition, a case of acute liver failure after withdrawal of chronic methotrexate use for RA has been reported [55]. However, these agents are considered as very low risk for HBV when used as monotherapy.

### Tumor Necrosis Factor-Alpha (TNF-α) Antagonists

Tumor necrosis factor-alpha (TNF-α) is a crucial pro-inflammatory and immunoregulatory cytokine in the pathogenesis of various inflammatory conditions. A number of anti-TNF-α agents are approved to treat conditions including RA, ulcerative colitis, Crohn’s disease, and psoriasis. TNF-α is also a critical cytokine in the coordination of innate and adaptive immune defense against HBV infection [56]. Blockade of TNF-alpha signaling can lead to increased HBV replication and reactivation. In a large retrospective analysis of 89 HBsAg-positive and 168 HBsAg-negative, anti-HBc positive patients treated with an anti-TNF agent, 35 HBsAg-positive patients (39 %) experienced HBVr, among whom five developed associated liver failure with death resulting in four [57]. Among the 168 HBsAg-negative, anti-HBc-positive patients, nine (5 %) experienced HBVr, with one case of fatal acute liver failure. Reactivation risk was higher with infliximab compared to other anti-TNF agents, and with concomitant use of other immunosuppressive drugs. In another investigation of 122 patients with HBV infection treated with anti-TNF agents, HBVr occurred in 15 (12.3 %), with etanercept implicated in ten cases and infliximab in two [10]. The U.S. Food and Drug Administration has issued a warning regarding HBVr with use of infliximab [58]. While these warnings represent an important first step, further investigation with regard to agent/indication and dose-specific risk is needed and should be collected in prospective, registered fashion to avoid case selection/reporting bias.

Prospectively performed studies have reported variable rates of activation with TNF-α use. In a small study, investigators followed 21 HBsAg-negative, anti-HBc-positive RA patients on TNF-α inhibitors and found no reactivation over 2 years [59]. In another study of 67 HBsAg-negative, anti-HBc-positive RA patients on therapy with an anti-TNF-α agent (23 infliximab, 23 etanercept, 19 adalimumab), there were no significant elevations of serum HBV DNA or appearance of HBsAg during mean follow up of approximately 4 years [60].

### Monoclonal Antibodies

Monoclonal antibodies, used to treat a wide variety of medical conditions [61], interfere with ligand binding cell surface receptors on either B or T cells, conferring immunomodulatory effects. Recent experience with regimens containing rituximab, a monoclonal antibody against the protein CD20 found on the surface of B cells, has demonstrated that when used in the treatment of malignancies in HBsAg-negative, anti-HBc-positive patients, there was an approximately six times higher odds ratio of HBVr compared to identical regimens without rituximab [12, 62]. A preliminary analysis of the post-marketing data from the FDA Adverse Event Reporting System found 109 cases of fatal HBV-related liver failure associated with rituximab or the anti-CD20 monoclonal antibody ofatumumab (Arzerra, GlaxoSmithKline, Research Triangle Park, NC); in more than half, screening was either inadequate (testing for HBsAg but not anti-HBc) or had not been done [63]. In September 2013, these findings prompted the FDA to add HBVr to the existing Boxed Warning of the Rituxan label, and to create a new Boxed Warning for the Arzerra label. In the *Warnings and Precautions* section of the labels for both drugs it is now recommended that before starting treatment all patients be screened by measuring HBsAg and anti-HBc; that when screening identifies patients at risk of HBVr, a hepatitis expert be consulted regarding monitoring and use of HBV antiviral therapy; that patients with evidence of prior HBV infection be monitored for clinical and laboratory signs of HBVr during therapy and for several months thereafter since reactivation has occurred up to 12 months after therapy completion with these drugs; that in patients who develop HBVr while on therapy, the drugs be immediately discontinued and appropriate treatment for HBV be started; and that any chemotherapy the patient is receiving be discontinued until the HBV infection is controlled or resolved [64•]. HBVr has also been reported in association with ibritumomab tiuxetan (Zevalin) [65], a CD20-directed radiotherapeutic antibody approved for treatment of B-cell non-Hodgkin lymphoma, and with alemtuzumab (Campath) [66], a monoclonal antibody directed against CD52 (expressed on B cells and T cells, natural killer cells, and macrophages) which is approved for refractory chronic lymphocytic leukemia [67]; although there are no current label warnings related to reactivation with these two drugs, physicians should be aware of the possibility.

### Systemic Chemotherapy

As discussed previously, HBVr has been studied most extensively in patients receiving treatment for lymphoma. In an early Asian study, 100 patients (27 HBsAg-positive; 51 HBsAg-negative/positive for anti-HBc and/or positive for anti-HBs; 22 negative for all three) had HBV DNA levels checked at baseline and prospectively followed [68]. HBVr-related liver failure occurred in 7 %, 2 %, and 0 % of patients, respectively. In another prospective study of 244 HBsAg-negative patients who received chemotherapy for lymphoma, eight developed reactivation (seven exposed to rituximab regimen), of whom three progressed to liver failure, one of whom died [69]. Combination regimens that contain anthracyclines, docetaxel or epirubicin have been linked to HBVr and death [43, 44]. HBVr has also been recognized with treatment regimens used for lung, colon, and liver malignancies [46, 70, 71].

### Transarterial Chemoembolization (TACE)

Transarterial chemoembolization (TACE), in which chemotherapeutic agents are administered into a branch of the hepatic artery, is used in treating hepatocellular carcinoma (HCC). TACE is widely used for “downstaging” tumors prior to liver transplantation [72, 73]. Reactivation of HBV replication has been reported in patients who have received TACE [74, 75]. In instances where supraselective arterial injection cannot be successfully accomplished or when there is inadvertent administration of the oncologic drug through arteriovenous shunts, systemic exposure has been shown to occur, partially accounting for the surprisingly high rate of reactivation in some case series [76••]. In a randomized controlled study of HBsAg-positive patients with HCC who received TACE with or without antiviral prophylaxis, HBVr was substantially higher in the group not receiving antiviral prophylaxis [74].

## Treatment Strategies

### Screening

A critical issue in the prevention of HBVr is the identification of those with HBV infection prior to initiation of immunosuppression. It estimated that in the U.S. less than one-third of patients with chronic HBV are aware of their status [77]. No validated screening tools have been routinely adopted into clinical practice; and studies suggest screening for HBV is greatly underutilized [78, 31]. Further complicating the issue is the lack of uniformity among major societies and their practice guidelines with regards to screening [79–82, 30, 83]. The development of appropriate screening tools and cost-effectiveness analysis studies are needed to determine the utility of universal vs. at risk screening before the use of immunosuppressive drug therapy.

### Antiviral Therapy (Prophylactic, Preventive, and Therapeutic)

Prophylactic antiviral therapy (therapy initiated prior to or concurrently with immunosuppressive therapy before an increase in viral replication or biochemical evidence of disease) has been demonstrated to greatly reduce although not completely eliminate HBVr and its sequelae. Antiviral therapy initiated as soon as HBV DNA and low level ALT increase are observed has also been suggested as a strategy in controlling HBVr. Five oral nucleos(t)ide analogue drugs are available for HBV treatment: lamivudine, adefovir, entecavir, telbivudine, and tenofovir [84]. Only lamivudine and entecavir have so far been studied as sole agents for either prophylaxis against or treatment of reactivation. In a review of 14 studies that compared HBsAg-positive cancer patients on chemotherapy given lamivudine as prophylaxis compared to patients not given prophylaxis, there was a much lower pooled incidence of reactivation in those on lamivudine (4 % vs 37 %) and fewer HBV-related deaths (2 % vs 7 %), with no patients on lamivudine developing HBV-associated liver failure compared to 13 % of those not given prophylaxis [85]. However, resistance and hepatitis flares have been reported in patients receiving preventive lamivudine during immunosuppressive therapy [86]. Entecavir and tenofovir are newer agents with potent antiviral activity; studies in immunocompetent patients with chronic hepatitis B have shown minimal resistance in treatment-naïve patients receiving entecavir and none with tenofovir [87, 88]. Entecavir as monotherapy has been shown to induce durable HBV DNA suppression in lymphoma patients treated with rituximab [40••, 89].

**Fig. 1:**
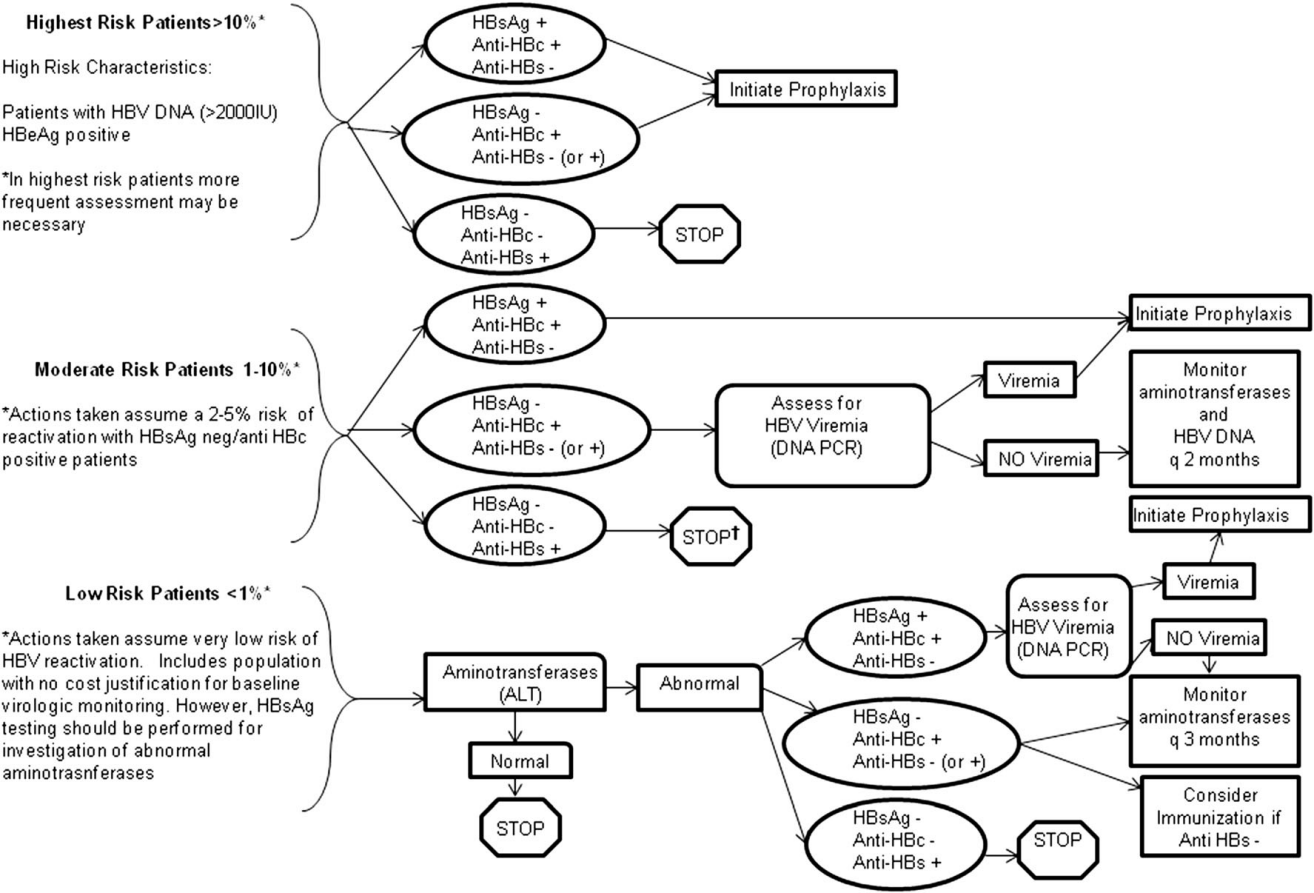
Proposed algorithm for HBV reactivation treatment and monitoring. Patients may be categorized into low, medium, or high risk dependent upon baseline characteristics and proposed agents. In medium and high risk populations serologic screening with HBsAg, anti-HBc, and Anti-Hbs should be performed. Serologic screening should be performed in those at low risk with unexplained abnormal aminotransferases † -In a patient found to be anti-HBs positive and anti-HBc negative on initial screen in a moderate risk setting, consider providing a dose of HBV vaccine (40 µg) as intermediate gesture and then stop

In two recent comparison studies, there has been a substantially reduced rate of HBVr in chemotherapy-treated lymphoma patients given entecavir prophylaxis (0 % [90] to 6.3 % [40••]) compared to those given lamivudine prophylaxis (12 % [90] to 39.3 % [40••]), as well as, in one study, markedly lower rates of hepatitis (6 % versus 27 %) and chemotherapy interruptions (6 % versus 20 %) [90]. In another recent study, among rituximab-treated lymphoma patients who received prophylactic entecavir (beginning prior to chemotherapy and continuing until three months after chemotherapy completion; n = 41) only one patient (2.4 %) experienced HBVr compared to seven patients (17.9 %) in the group chosen to receive only therapeutic ETV at the time of HBVr (n = 39) [91•]. In light of these and other studies, entecavir appears to be a favored agent for HBVr prophylaxis or therapy [92, 93], in large part because with long-term use there may be resistance-associated failures of lamivudine. While studies with tenofovir have not yet been reported in this clinical situation, it is anticipated that its use would be as effective for prophylaxis as entecavir. While prophylactic antiviral therapy clearly appears to substantially lower the risk of HBVr and can be applied in outpatient settings [94], the cost effectiveness of this approach is dependent upon the clinical population it is applied to and agent specific risk of reactivation. For example, while a strong case can be made for prophylactic therapy in HBsAg-positive patients treated with rituximab, this approach would almost certainly not be cost effective in patients who have isolated anti-HBc and are placed on a single agent (for example, azathioprine) with limited immunosuppressive potency (Fig. 1).

### Therapy Cessation

While data supports the use of prophylactic therapy as well as the use of the newer nucleos(t)ide analogues compared with lamivudine, less is known about when therapy can be safely withdrawn. In one study of 80 HBsAg-negative, anti-HBc-positive patients with lymphoma randomly assigned to entecavir prophylaxis or no prophylaxis (control), patients in the control group developed reactivation as late as 17 months after the start of rituximab, or approximately 11 months after the cessation of rituximab therapy [95]. High levels of serum HBV DNA (≥4 log10 copies/ml) before chemotherapy predicted HBVr after withdrawal of prophylactic antiviral therapy. Some experts feel that antiviral treatment should generally be continued for six months after immunosuppressive drug therapy is discontinued and for 12 months when rituximab is used or whenever HBV DNA above 2000 IU or 10,000 copies/mL is observed at baseline [96]. Alternatively, while more challenging, case-by-case decisions may need to be made with consideration of both the potency and therapeutic half-life of the agent being used as well as serologic and virologic status of the patient; for example, the presence of sustainable high titer anti-HBs (>100 IU) on immunosuppressive treatment requiring shorter term prophylaxis and the presence of higher levels of HBV DNA at baseline requiring longer periods of treatment/prophylaxis.

## Conclusions

Reactivation represents a loss of immune control in those chronically infected with or previously exposed to HBV. This loss of control is often caused by immunosuppressive therapies through various direct and indirect mechanisms. Immunosuppressive therapy has revolutionized the treatment of many disorders. New cytokine inhibitors such as anti-IL-17 [97] and anti-IL-23 [98] are being tested in a variety of clinical settings. As the use of immunosuppressive agents increases, so too will the incidence of HBVr and associated complications. Of critical importance is screening at-risk populations for the presence of infection or previous exposure. Multidisciplinary, randomized controlled studies are needed to formulate a framework for risk stratification based on patient and viral factors as well as disease indication and immunosuppressive agent.
